# Opening Flask, Packing, Vulcanizing

**Published:** 1907-01

**Authors:** Stewart J. Spence

**Affiliations:** Chattanooga, Tenn.


### The American Journal of Dental Science

The Official Organ of the Florida, Georgia and South Carolina State Dental Societies

Third Series D 225

VOL. XXXVIII JANUARY, 1907 NO. 1

EDITORS

WM. GIRD BEECROFT, D. D. S., Madison. Wis. B. H. TEAGUE, D. D. S., Aiken, S. C.

Published on the 10th day of each month.
Subscription price $1.00 per year.
ENTERED AS SECOND-CLASS MATTER, MADISON, WIS.

Address all communications, both business and editorial, to
**The Dental Publishing Company, Madison, Wisconsin**

### OPENING FLASK, PACKING, VULCANIZING

STEWART J. SPENCE, CHATTANOOGA, TENN.

These papers proceed under the supposition that the base-plate is to be vulcanite. I digress briefly, however, to say of celluloid and aluminum:

Many years ago the writer discontinued the use of celluloid for base, believing it inferior to rubber because (1) softer and weaker, and (2) it absorbs oval fluids and thus becomes indelibly stained. If any one cares for my experiments and directions for using this material I refer them to my article upon it in Richardson's *Mechanical Dentistry*.

On the contrary, aluminum for base is to be recommended. It is lighter, stronger, cleaner, and a better conductor than vulcanite and does not contract, like it, in cooling. Besides, some high authorities, as Dr. Haskell, contend that the mercuric salt used in coloring is with persons injurious to health. This difficulty, however, may be met by using black rubber. If swaging be done by screw-pressure the plate will sit perfectly "dead" in the die, entirely without the "spring" which is so apt to come from hammer swaging, but with more spread of the aluminum. Dr. P. B .McCullough of Philadelphia is bringing out a swaging device which appears to be the latest advance in that line, substituting for the old-time counters of lead, of shot, of modelling compound, a moist putty which he terms plasm, this being prevented from oozing out by his device, and by its plasticity readily conforming to all parts of the die. For the latter he uses plaster of paris or Spence's Plaster.

To return to vulcanite: The writer prefers hot water to dry heat in opening and closing flask. He picks out what wax he can and washes away the balance by poured hot water. A little wax may remain without harm, as it does not, as does oil, injure rubber.

If plain teeth and pink rubber gums are used, some care is needed to prevent the red or black rubber from appearing in places through the pink, especially at interdental spaces. These spaces should be packed firmly with V-shaped pieces of pink rubber, two pieces usually to each space, and then two strips of pink should be stretched over said spaces and as near to the pins as dare be without touching. For strength's sake, pink rubber should not touch pins. If placed near and above them, compact is not apt to occur because the rubber tends to flow, if at all, outward, and thus upward. For this reason, an excess of red rubber about the rug and pins is apt to squeeze under and force upward the pink, showing through at interdental spaces. Avoid such excess.

While the practice of using nothing but pink rubber for gums in front is to be condemned because of weakness, yet if only one thickness of pink be laid on, the dark rubber beneath is apt to show through enough to give a cloudy appearance to the gums.

To find the required amount of rubber, take the wax that has come out of flask, and place it in a narrow, tall glass, weighting it to make it sink in the water. The glass should have been marked by a scratch near its rim, made by a diamond or emery wheel. With syringe add or subtract water until it is level with this scratch. Then remove the wax and put in the rubber until it brings the water to same level, *and a little above it,* because some wax is lost in melting and sometimes spaces exist between the model and wax trial-plate.

But be careful not to have much excess of rubber, and cut waste gates freely, because as the closure of the flask proceeds the imprisoned rubber becomes dense and hard from compression and—especially in the last turn of the screw, when the pressure is enormous,—reacts on the model, compressing it out of shape to an extent hardly credible by those who have not tested it. Here again Spence's Plaster, because of its great hardness, is indicated. If the flask closes too easily, open and add more rubber; if too hard, open before fully closing and remove some of the rubber.

In my last paper I dwelt on the subject of the contraction of rubber; it remains only to say that in packing the flask thick portions of the flask should be treated to prevent vertical contraction. I have employed notched strips of metal stuck nearly vertical into the rubber, but prefer my non-contracting rubber (also non-porous), which consists in one part by weight of pulverized aluminum and three parts rubber; the latter being softened by chloroform and the former macerated into it. Weighted rubber, because already containing metal, requires but one-half as much aluminum, and even when used as it is it reduces contraction about 50 per cent.

Vulcanization by dry heat is surrounded by difficulties, because the heat will not ascend above 212 degrees until the water in the plaster is evaporated; after which it goes up rapidly and is hard to control. But for this difficulty, dry vulcanization would have considerable advantage over wet, viz., that it does not, as does the wet, cause swelling of the plaster of Paris, but even slightly contracts it. The expansion of a plaster of Paris model during wet vulcanization (or rather, during its earliest stages) is about equal to that which occurs during its crystallization. Here again Spence's Plaster is of value, having no such expansion.

Every vulcanizer ought to be equipped with a Snow Regulator; not only because this prevents too rapid heating, too high heating, and too long heating, but rather because it removes the necessity for watching the vulcanizer, and—most important—the danger of explosion from neglect in watching.

Black rubber, because of its superior strength and elasticity, ought to be used in preference to red, except with patients who expose to view the lingual surfaces, which is especially apt to occur with the lower plate about the bicuspids. But better than red for this purpose, is a veneer of pink rubber, and this cut away as much as possible from the lingual surfaces of the teeth. The black rubber indicated is not that called "jet black," which is colored artificially, and of course weakened thereby, but the natural black. This last is somewhat more troublesome to pack than rubbers loaded with foreign rubber, and does not spread so easily in flask-closing, being therefore more liable to fail to reach all parts of the matrix, and also more apt to compress the model, if of plaster of Paris, but its contraction in cooling is very little more than red rubber, it is considerably stronger, its greater porosity can be overcome by using the before mentioned aluminumized rubber to fill in the thick parts, black makes a handsome contrast with pink, and if the coloring matter of red rubber is poisonous, this evil is avoided.

